# Comparative proteomic analysis of the membrane proteins of two *Haemophilus parasuis* strains to identify proteins that may help in habitat adaptation and pathogenesis

**DOI:** 10.1186/1477-5956-12-38

**Published:** 2014-07-07

**Authors:** Luhua Zhang, Yiping Wen, Ying Li, Xingliang Wei, Xuefeng Yan, Xintian Wen, Rui Wu, Xiaobo Huang, Yong Huang, Qigui Yan, Mafeng Liu, Sanjie Cao

**Affiliations:** 1 Key Laboratory of Animal Disease and Human Health of Sichuan Province, College of Veterinary Medicine, Sichuan Agricultural University, Ya’an, Sichuan 625014, PR China

**Keywords:** Comparative proteomics, *Haemophilus parasuis*, Habitat adaption, Pathogenesis

## Abstract

**Background:**

*Haemophilus parasuis* is the causative agent of Glässer’s disease characterized by polyserositis, arthritis, and meningitis in pig, leading to serious economic loss. Despite many years of study, virulence factors and the mechanisms of the entire infection process remain largely unclear. So two-dimensional gel electrophoresis and mass spectrometry were used to search for distinctions at the membrane protein expression level between two *H. parasuis* isolates aimed at uncovering some proteins potentially involved in habitat adaption and pathogenesis.

**Results:**

A comparative proteomic approach combining two-dimensional gel electrophoresis with mass spectrometry and tandem mass spectrometry was employed to explore the differences among membrane proteomes of a virulent *Haemophilus parasuis* strain isolated from the lung of a diseased pig and an avirulent strain isolated from the nasal swab of a healthy pig. Differentially expressed protein spots identified by mass spectrometry were annotated and analyzed by bioinformatic interpretation. The mRNA level was determined by quantitative real-time PCR. Proteins representing diverse functional activities were identified. Among them, the tonB-dependent siderophore receptor was a new discovery highlighted for its activity in iron uptake. In addition, periplasmic serine protease and putrescine/spermidine ABC transporter substrate-binding protein were given focus because of their virulence potential. This study revealed that the differentially expressed proteins were important in either the habitat adaption or pathogenesis of *H. parasuis*.

**Conclusions:**

The outcome demonstrated the presence of some proteins which raise the speculation for their importance in helping in habitat adaption or pathogenesis within the host.

## Background

*Haemophilus parasuis* is a pleomorphic, Gram-negative, nicotinamide adenine dinucleotide-dependent rod belonging to family *Pasteurellaceae*. This bacterium is the causative agent of Glässer’s disease in pig characterized by polyserositis, arthritis, and meningitis [1], leading to serious economic loss [2]. Fifteen *H. parasuis* serotypes have been described to date. Among them, serotype 4 is prevalent in field strains in most countries, and serotype 3 is the predominant strain isolated from the upper respiratory tract of healthy animals [3-5]. Different serotypes show significant differences in virulence, and variations have also been reported within the same serotype [6,7].

Several virulence factors contributing to the pathogenesis of Glässer’s disease have been reported in virulent strains, including lipooligosaccharide [8], polysaccharide biosynthesis protein [9], outer membrane protein P2 [10], cytolethal distending toxin [11,12], extracellular serine protease [13], autotransporters [14,15], capsule [16], hemolysin [17], sialylation [18], UDP-glucose pyrophosphorylase, and UDP-glucose 4′-epimerase [19]. Meanwhile, many of them can also be detected in avirulent strains.

*H. parasuis*, including virulent strains, is a ubiquitous bacterium in the upper respiratory tract of healthy pigs in most farms [20]. Only under certain conditions do the virulent strains invade a host to cause severe disease. Therefore, the phase of colonization and adaptation to the upper respiratory tract plays a crucial role in the entire infection of *H. parasuis.* Despite many years of study, the mechanisms of the entire infection process including colonization, invasion, and pathogenesis remain largely unclear, especially survival in the upper respiratory tract.

Most studies on the pathogenicity of *H. parasuis* are about genetic characteristics, and the expression level of related proteins is often ignored. Zhou et al. [21] provided systematic reference maps of outer membrane, intracellular, and extracellular proteome fractions, which can serve as an important resource for studying the pathogenesis of *H. parasuis*. However, no other comparative proteomic studies have been performed. Therefore, research on the proteins related to adaptation and pathogenesis would further our understanding of the survival mechanism of *H. parasuis*.

Proteomics is a powerful platform for revealing factors involved in pathogenicity and multi drug resistance in bacterial pathogens [22-24]. Proteomic analysis of membrane proteins has already been reported in some pathogenic microorganisms [25-27]. In our study, two *H. parasuis* isolates were selected, i.e., the virulent strain SC-1 (serotype 4; isolated from the lung of a diseased pig) and the avirulent isolate SC105 (serotype 3; isolated from the nasal swab of a healthy pig). A comparative proteomics method combining two-dimensional gel electrophoresis (2-DE) and mass spectrometry (MS) were used to search for distinctions at the membrane protein expression level between the two isolates aimed at uncovering some proteins potentially involved in habitat adaption and pathogenesis.

## Results and discussion

### Virulence comparison between *H. parasuis* strains SC-1 and SC105

In our study, to reveal some proteins that may participate in pathogenesis in *H. parasuis*, two virulent differentially isolates SC-1 and SC105 were selected for comparative proteomic analysis. For confirming the differences in virulence, the two strains were used for challenge experiments in mice. At 1 week post-infection with SC-1, the average mortality for mouse intraperitoneally injected with 8 × 10^8^, 1.1 × 10^9^, 1.4 × 10^9^, 1.7 × 10^9^, and 2.0 × 10^9^ cfu/mouse, was 0% (0/10), 20% (2/10), 50% (5/10), 70% (7/10), and 100% (10/10), respectively. All groups of mice injected with strain SC105 exhibited no clinical symptoms or gross lesions at necropsy. In the PBS control group, no anomalies were observed throughout the entire virulence experiments. The 50% lethal dose (LD50) value of strain SC-1 was calculated to be 1.4 × 10^9^ cfu/mouse. Based on the results, strain SC-1 was confirmed to be pathogenic and virulent and strain SC105 was regarded as avirulent.

### 2-DE and protein identification

**Figure 1:**
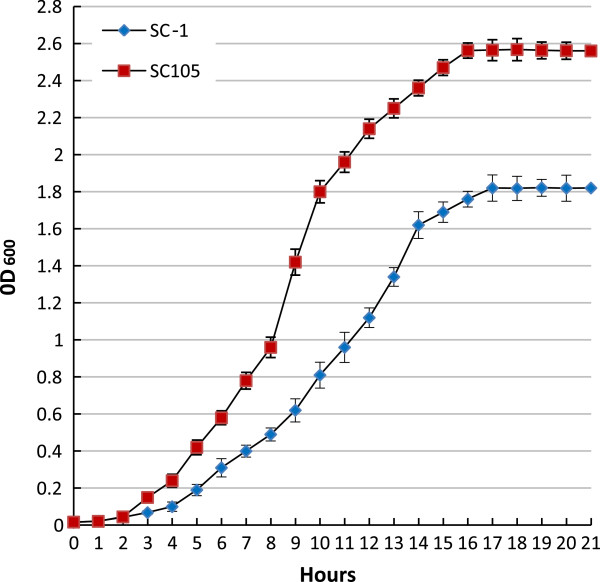
**Growth curve of *H. parasuis *strain SC-1 and SC105.** The cells were harvested at the early exponential growth phase of the cultures.

**Figure 2:**
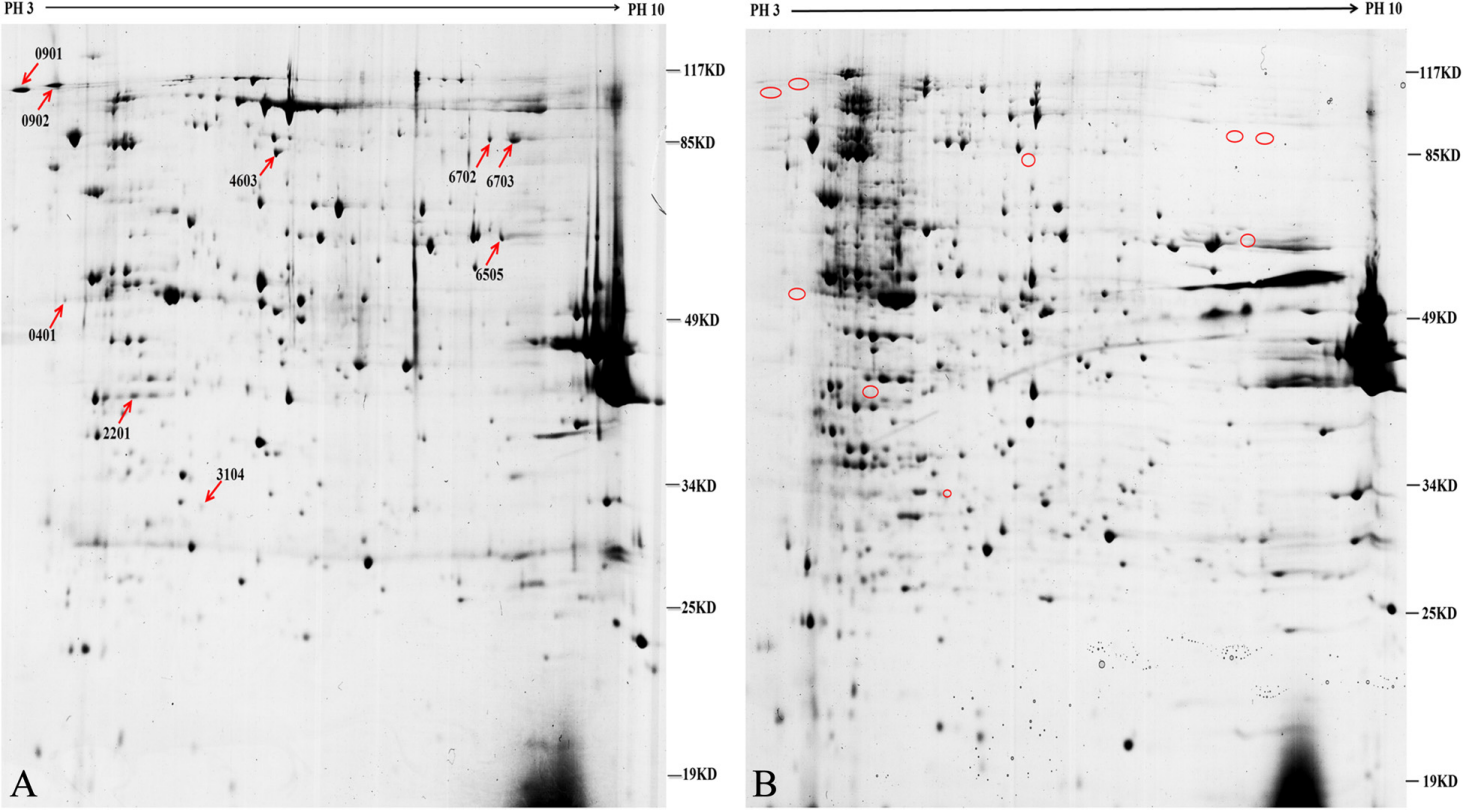
**Comparative analysis of the membrane proteome of *H. parasuis *strain SC105 (A) and SC-1 (B).** The differentially expressed proteins in strain SC105 versus SC-1 are marked by red arrows and locus numbers. Molecular weights are indicated on the right.

**Figure 3:**
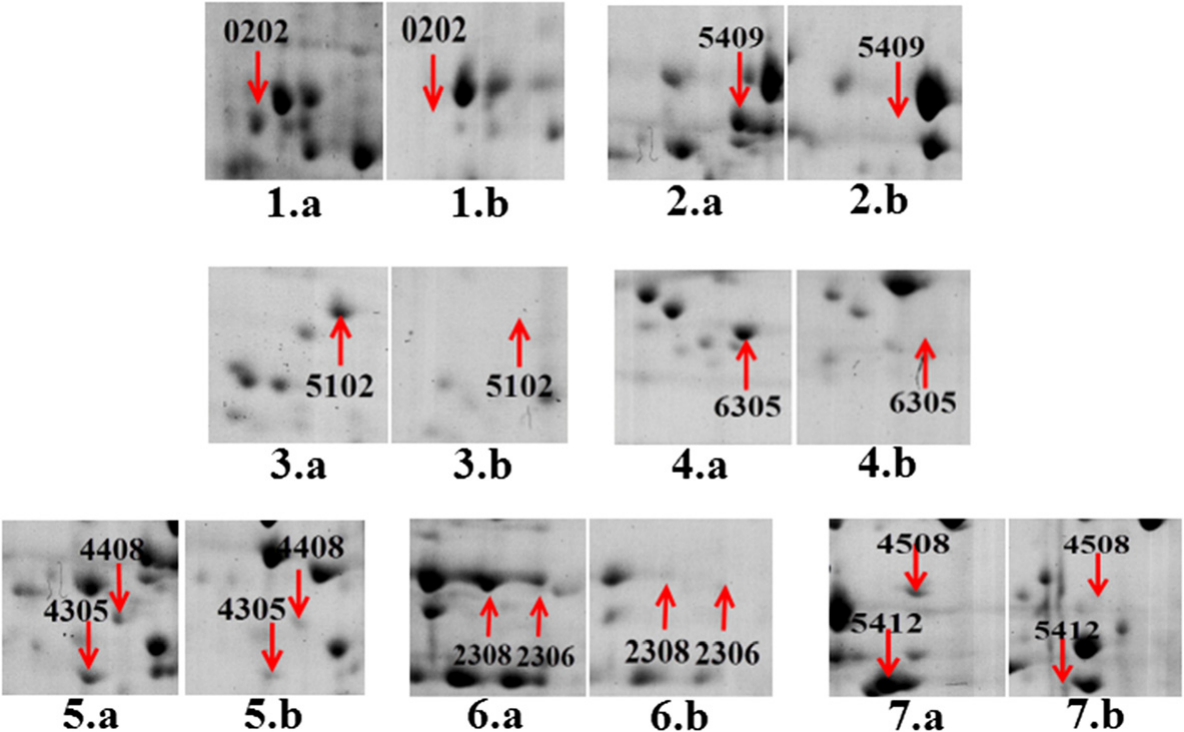
**Comparative analysis of the membrane proteome of *H. parasuis *strain SC-1 (a) and SC105 (b).** 1,2,3,4,5,6,7-(a) enlarged 2DE gel images of over expressed proteins in strain SC-1 that were absent in SC105 1,2,3,4,5,6,7- (b).

For proteomic analysis, the cells of strains SC-1 and SC105 were harvested at 8–10 h post- subcultivation, which was the early exponential growth phase of the cultures (Figure 1). Membrane proteins were separated with 17 cm IPG strips of pH 3–10. Approximately 189 ± 15 protein spots with molecular weights between 19 and 117 kDa were detected in each gel of strain SC-1, whereas approximately 191 ± 16 protein spots were detected in each gel of strain SC105. Further analysis showed nine protein spots in SC105 gels but not in SC-1 gels (Figure 2). Meanwhile, 10 protein spots were detected in SC-1 gels but not in SC105 gels (Figure 3). All 19 differential protein spots were confirmed when they were present in all three replicate gels. The selected protein spots were then characterized by MALDI-TOF MS and MALDI-TOF MS/MS. Nineteen protein spots corresponding to 17 individual proteins were successfully identified and are listed in Tables 1 and 2. However, the experimental data revealed tonB-dependent siderophore receptor family protein and periplasmic serine protease do/hhoA-like protein identified from two different positions in the same gel. They differed in either the Mw or pI value or both. The reason was most likely post-translational proteolytic modification or the phosphorylation of multiple residues. Similar results have been previously reported [21,28].

**Table 1:** Summary of differentially expressed proteins in SC105 versus SC-1

**Locus name**	**Accession number**	**Description**	**MW**^**a**^**/P** ***I***^**b**^	**Protein score**	**Total ion score**	**Localization**	**Classification**
SSP 0901	gi|544679443	outer membrane autotransporter protein	100488.7/4.74	415	312	multiple localization sites	Autotransporter
SSP 0902	gi|529485332	putative extracellular serine protease, EspP	61030/5.84	293	170	Unknown	Unknown
SSP 0401	gi|529260020	putative uroporphyrin-III C-methyltransferase, hemX	50714.1/4.62	537	362	CytoplasmicMembrane	Unknown
SSP 2201	gi|529486640	formate dehydrogenase iron-sulfur subunit,fdxH	34673.6/5.18	310	213	CytoplasmicMembrane	Energy metabolism
SSP 3104	gi|544684696	1-deoxy-D-xylulose 5-phosphate reductoisomerase, dxr	43367.5/5.3	379	195	Unknown	carbohydrate metabolism
SSP 4603	gi|544678965	2′,3′,-cyclic-nucleotide 2′,-phosphodiesterase	72641.3/6.19	230	78	multiple localization sites	nucleotide metabolism
SSP 6505	gi|529258413	heme-binding protein A, HbpA	59454.1/7.1	370	215	Periplasmic	transporter
SSP 6702	gi|529260706	tonB-dependent siderophore receptor family protein	79102.7/8.15	467	228	OuterMembrane	transporter
SSP 6703	gi|529260706	tonB-dependent siderophore receptor family protein	79102.7/8.15	387	133	OuterMembrane	transporter

^a^MW: Molecular Weight. ^b^P*I*: Isoelectric Point.

**Table 2:** Summary of differentially expressed proteins in SC-1 versus SC105

**Locus name**	**Accession number**	**Description**	**MW**^**a**^**/P** ***I***^**b**^	**Protein score**	**Total ion score**	**Localization**	**Classification**
SSP 0202	gi|506420014	putrescine/spermidine ABC transporter substrate-binding protein, PotD	39275/5.13	65	55	Periplasmic	transporter
SSP 2306	gi|498484268	phosphoglycerate kinase, pgk	41488.9/5.15	222	53	Cytoplasmic	carbohydrate metabolism
SSP 2308	gi|491996996	2-dehydro-3-deoxyphosphooctonate aldolase, kdsA	31563.9/5.36	213	77	Cytoplasmic	cell envelope
SSP 4305	gi|529270383	periplasmic serine protease do/hhoA-like protein, htrA	48120.4/6.82	261	123	Periplasmic	protein fate
SSP 4408	gi|514061512	S-adenosylmethionine synthetase, metK	42167.4/5.74	265	111	Cytoplasmic	amino acid metabolism
SSP 4508	gi|529266372	glutathione reductase, gor	49689.4/6.03	279	130	Cytoplasmic	amino acid metabolism
SSP 5102	gi|529262591	omp26 outer membrane protein, HlpA	29730.8/8.41	537	315	Unknown	Chaperones
SSP 5409	gi|529270383	periplasmic serine protease do/hhoA-like protein, htrA	48120.4/6.82	356	175	Periplasmic	protein fate
SSP 5412	gi|529272906	cysteine desulfurase, iscS	45398.1/5.91	576	369	Cytoplasmic	amino acid metabolism
SSP 6305	gi|529266699	phospho-2-dehydro-3-deoxyheptonate aldolase, aroF	39119.1/6.56	212	116	Cytoplasmic	amino acid metabolism

^a^MW: Molecular Weight. ^b^P*I*: Isoelectric Point.

The secretion and location of the identified proteins were predicted by various bioinformatics software packages. Five proteins (spot 6505, spot 6702/6703, spot 0202, spot 4305/5409, and spot 5102) were predicted to contain signal peptide, indicating that they were secreted through the classical secretory pathway. Among these five proteins, no lipoprotein was predicted. Another five proteins (spot 0901, spot 0902, spot 0401, spot 2201, and 4603) were identified as non-classical secreted proteins. Spot 0401 was the only protein identified to have one transmembrane domain. The results of cellular localizations and functional categories are summarized in Tables 1 and 2.

### Verification of protein expression by gene transcriptional expression

**Figure 4:**
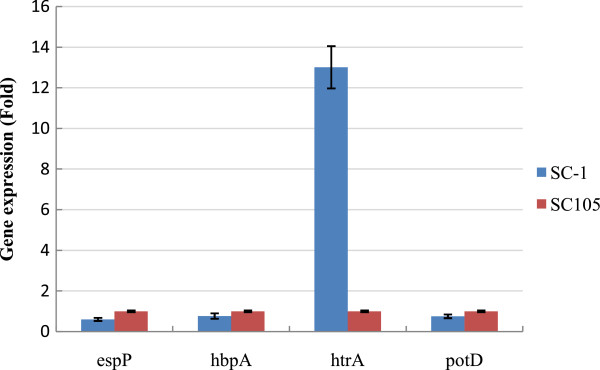
**Quantitative RT-PCR analysis of four gene transcripts.** The x-axis represents the four genes of *H. parasuis* and the y-axis represents the gene expression level. Samples were normalized to the 16S rRNA gene as a control. The average values and standard deviations in three quantitative PCR analyses are shown.

To validate the proteomics data, we performed qRT-PCR to examine the mRNA expression levels of selected genes with differences at the protein level taking 16 s rRNA as the reference gene. Results showed that qRT-PCR data generally corresponded with previously obtained data by 2-DE PAGE, with the exception of gene *potD* (Figure 4). In the case of periplasmic serine protease (HtrA), qRT-PCR results showed its obvious higher level of transcription in SC-1 (virulent strain) than in SC105 (avirulent strain), indicating that HtrA was an important virulence attribute. The mRNA levels of both *hbpA* and *espP* genes revealed slightly higher expression level in SC105 versus SC-1 in heme-binding protein and putative extracellular serine protease, respectively.

Comparison between tanscriptome and proteome data sometimes yielded a low correlation [29,30]. The low correlation between mRNA and protein levels due to substantial posttranscriptional regulation [31]. Using transcriptomic and proteomic data, Wu et al. analyzed the posttranscriptional regulation in the Yeast *Saccharomyces cerevisiae*, and found that all posttranscriptional biological properties contributed to about one-third of the total variation of mRNA–protein correlation [31]. In our study, we tried our best to perform uniformly as described by Methods. Unexpectedly, the transcription level of gene *potD* in SC105 was slightly higher than in SC-1, which was inconsistent with the protein expression level in the two strains. Regarding the inconsistency between transcription patterns and protein-synthesis patterns, we speculated that this discrepancy might be influenced by posttranscriptional regulation. In addition, some significant factors including protein degradation, codon and amino acid composition and translation elongation also contributed to the inconsistency between mRNA and protein levels of gene *potD*[31].

### Analysis of differentially expressed proteins

Membrane proteins account for about 20% to 30% of all predicted proteins [32] and play crucial roles in numerous cellular processes, including nutrient and metabolite transport, energy metabolism, and expulsion of toxins and antibiotics. Thus, membrane proteins are indispensable for adaptation and survival in the environment [33]. Additionally, membrane proteins are significant in the course of infection for direct contact with host cells [12,34]. Therefore, membrane proteins of *H. parasuis* were chosen to reveal its survival mechanism.

### Proteins associated with habitat adaptation

The nutritional deficiency and a strong defense system in the upper respiratory tract are significant challenges for various microorganisms to survive. However, microbes have developed diverse and elaborate systems to obtain essential nutrient and escape from immune clearance, ready for invasion. An epidemiological investigation has shown that *H. parasuis* is a ubiquitous bacterium in the upper respiratory tract of healthy pigs [20]. Under certain conditions, virulent strains can invade a host and lead to severe disease. However, the mechanisms of *H. parasuis* survival in the upper respiratory tract are largely unknown.

In the present study, we selected strain SC105 identified as serotype 3 as a research sample. Previous studies have shown that serotype 3 strains are the predominant strains isolated from the upper respiratory tract of healthy animals [3-5]. The other strain, SC-1, was isolated from the lung of a diseased pig. Studies comparing the representative strains, i.e., serotype 3 strains, in the upper respiratory tract with strains from other places may play an important role in uncovering the mechanism of habitat adaptation. Proteins that are considered to be consistent with the adaptation to the upper respiratory tract, however, are not necessarily unrelated with the virulence factors and pathogenesis of *H. parasuis*. In the current work, eight differentially expressed proteins unique to SC105 are marked in Figure 2, among which four proteins most likely related to habitat adaptation are introduced in detail.

TonB-dependent siderophore receptor family protein (spots 6702 and 6703), over expressed in SC105, is composed of a membrane-spanning beta barrel with an N-terminal “plug” domain from the periplasmic end of barrel that completely occludes the barrel’s interior [35]. The siderophore binds to a binding pocket formed by a cluster of extracellular looped together with several residues from the plug domain with a high affinity. After this event, the tonB-dependent energy system induces conformational changes of the plug domain, i.e., either a channel sufficiently large to allow transport is created, or the ligand is directly driven into the periplasm [36]. Thus, this protein is involved in the acquisition of iron from the surrounding environment, which is recognized as one significant factor in the successful establishment of the pathogen in the host. Until recently, the complete genomes for 10 isolates of *H. parasuis* were sequenced [37]. MASCOT was used to search the data against the NCBInr protein database, and spots 6702 and 6703 matched the tonB-dependent siderophore receptor family protein in *H. parasuis* (Figure 2). Further BLAST showed that tonB-dependent siderophore receptor was identified in the newly sequenced serovar 3 strain SW114 and serovar 9 strain D74 of *H. parasuis*. As shown previously, serovar 3 strains were predominantly isolated from the upper respiratory tract [5], but serovar 9 strains were isolated from cases with systemic disease (polyserositis, arthritis, and meningitis) [6]. The available data showed that the new identified tonB-dependent siderophore receptor may contribute to adaptability of *H. parasuis* to both respiratory and systemic sites.

To our knowledge, this report is the first to identify another tonB-dependent siderophore receptor in *H. parasuis*, which has low sequence identity (21.2%) with the previously reported ferric hydroxamate uptake protein FhuA [38]. However, *H. parasuis* does not possess genes that encode proteins associated with siderophore synthesis [39]. Thus, we speculated that the newly identified siderophore receptor may enable the utilization of several siderophores from other organisms in the upper respiratory tract colonized by *H. parasuis*. Similar studies have been previously reported [40-42]. This strategy for iron exploitation is consistent with the adaptability of bacteria to the iron-poor environment in the host.

Heme-binding protein A (HbpA; spot 6505), an up-regulated protein in SC105, has been discovered in *Haemophilus influenza* and turned out to be conserved among heme-dependent *Haemophilus* species [43]. The protein was considered to be responsible for the import of heme into an organism and a crucial element for the survival of the bacteria in host [44,45]. In addition, the protein has also been identified as a virulence determinant [46]. However, Vergauwen et al. [47] recently reported that the dominant function of HbpA is glutathione import rather than heme utilization, both of which are actively involved in bacterial adaptation to a new environment to multiply in a short time. Anyway, these study findings suggested that the HbpA may be important in habitat adaptation. HbpA has also been identified in serotype 5 strain SH0165 studied by Zhou et al. [21]. This finding may indicate that HbpA was also a putative virulence associated factor in *H. parasuis*.

Outer membrane autotransporter protein (AidA; spot 0901), namely, “adhesin-involved-in-diffuse-adherence” and putative extracellular serine protease (EspP; spot 0902), a member of the serine protease autotransporters of Enterobacteriaceae, both are over expressed in SC105 belonging to the simplest protein secretion system type V. Three structural motifs, i.e., a signal sequence, an N-terminal passenger domain, and a highly conserved channel-forming C-terminal domain are contained as basic components of AidA and EspP proteins [48]. Autotransporter proteins are always associated with virulence functions, such as adhesion, biofilm formation, aggregation and so on. The passenger domain of autotransporter AidA remained non-covalently bound to the bacterial surface after cleaved as adhesion to interact with the host cells [48]. This enables the bacteria to colonize in the host by adhering to host cell surfaces or the extracellular matrix. EspP is a protease autotransporter released from the bacterial cell into the external milieu that can cleave persin A and human coagulation factor V [49,50]. And Dziva et al., [51] showed that EspP is involved in the intestinal colonization and adherence to bovine primary intestinal epithelial cells. However the mechanism is still unclear. Studies have shown that AidA and EspP proteins are actually virulence factors in many other microorganisms and display enhanced pathogenicity [49,51-53]. Therefore, important roles of AidA and EspP proteins may play in the colonization and adaptation of *H. parasuis*, a ubiquitous bacterium in the upper respiratory tract of the host, merit further investigation.

### Proteins associated with pathogenesis

A comparison of membrane proteins between the virulent strain SC-1 and the avirulent strain SC105 was performed to reveal virulent proteins crucial to pathogenesis. Ten protein spots corresponding to nine individual proteins differentially expressed in SC-1 were successfully identified and marked in Figure 3. Three of the identified proteins, i.e., periplasmic serine protease do/hhoA-like protein, putrescine/spermidine ABC transporter substrate-binding protein, and 2-dehydro-3-deoxyphosphooctonate aldolase were suspected to be virulence factors. However, to our knowledge, no study has reported about the virulence potential of these proteins in *H. parasuis*.

Periplasmic serine protease (HtrA; spots 4305 and 5409), also known as DegP or Do protease, is a heat shock-induced and ATP-independent protease with homologs in a wide range of bacteria and eukaryotes. In our study, the data showed that HtrA protein was over expressed in both mRNA and protein levels in virulent strain SC-1. The defining feature of the HtrA family is the combination of a proteolytic domain with at least one C-terminal PDZ domain, which may be involved in the binding of substrate or effector. HtrA proteins are typically active in various aspects of protein quality control, including protease to degrade the abnormal cell envelope proteins and chaperones to promote folding or to protect substrates from proteolytic degradation [54]. DegP reportedly chaperones the extended filamentous hemagglutinin polypeptide in the periplasm and is thus involved in the secretion of virulence factors of *Bordetella pertussis*[55]. In addition, HtrA can reportedly cleave the ectodomain of the cell-adhesion protein E-cadherin as a secreted virulence factor in *Helicobacter pylori*[56]. The high attenuation and decreased survival in a mouse model of *htrA* mutant in *Salmonella typhimurium* and *Brucella* provide a good indication of its virulence association [57]. The important role of HtrA in protein fate and cell physiology may implicate its involvement in bacterial pathogenesis and virulence.

Putrescine/spermidine ABC transporter substrate-binding protein (PotD; spot 0202), which is involved in polyamine transport, is encoded by *potD* gene. Differential expression of this protein in SC-1 and SC105 is shown in Figure 3. In *Streptococcus pneumonia,* protein PotD is the polyamine-binding protein of the PotABCD system. In addition, PotA is an ATPase and PotBC are integral membrane proteins that form a polyamine-specific transport channel [58]. Polyamine transport systems may play an important role in *S. pneumonia* pathogenesis, especially under physiological stress [59]. Given the particular importance of polyamines in the cellular processes of transcription and translation in the initial steps of pneumococcal infection, PotD is considered to be potentially associated with virulence in *S. pneumonia*, which has been identified in murine models of systemic and pulmonary infection [58]. PotD has also been proposed to be virulence-related proteins in *Aggregatibacter actinomycetemcomitans*[60]. Furthermore, Zhang et al. [61] shows that PotD may change the rate of polyamine synthesis and stimulate the biofilm formation in *Escherichia coli*, which emphasizes the importance of PotD as a potential virulence factor.

2-Dehydro-3-deoxyphosphooctonate aldolase (KdsA; spot 2308), namely, KDO-8-phosphate synthase, an up-regulated protein in SC-1, catalyzes the first step in the biosynthesis of 3-deoxy-D-manno-octulosonic acid (KDO), which is involved in the connection of the lipid A moiety and the oligosaccharide chain in lipooligosaccharide (LOS) molecule. A variety of glycosyl transferases sequentially added the core and branch oligosaccharide to the acceptor, the lipid A-KDO molecule, and extensions for the LOS molecule [62]. As a major surface-exposed constituent of the outer membrane, the LOS molecule mediates adhesion onto porcine brain microvascular endothelial cells and newborn pig tracheal cells and can also induce the release of IL-8 and IL-6 by both cells in *H. parasuis*[63,64]. The importance of the LOS molecule in the pathogenesis of infection may also make KdsA a possible virulence-associated factor and an attractive target for antibiotic treatment.

### Proteins as novel vaccine targets

Membrane proteins are the most promising vaccine candidates because of their significance in the course of infection and invasion for direct contact with host cells [65]. In this study, three proteins, outer membrane proteins 26 (Omp26), PotD and HbpA were proposed to be potential vaccine candidates based on the related literature.

Omp26, a 26 kDa protein in nontypeable *H. influenza* (NTHI), can be a suitable vaccine candidate against NTHI infection because of its capacity to induce high levels of specific antibodies in the serum of immunized rats and enhance bacterial pulmonary clearance [66]. Recently, Omp26 of NTHI as an antigen was harboured in *E. coli* ghosts and was applied to vaccinate in a rat model. The results of this study showed that mucosal immunization with recombinant Omp26 can induce a specific and protective immune response [67]. Omp26 of *H. pylori* has also been expressed with *Mycobacterium fortuitum* β-lactamase protein as a fusion, whose induced strong protection shows that Omp26 is a potential vaccine candidate antigen for therapy against *H. pylori*[68]*.*

The immunoprotection of recombinant PotD was confirmed by Shah et al. [69] in *S. pneumonia*, and its role as a potential vaccine candidate was proposed in *Actinobacillus pleuropneumoniae*[70]. However Chen et al. [71] showed that PotD has lower or no protection against infection in a swine model, even though they induced high levels of antibodies. They also found that recombinant protein HbpA can induce high titers of antibodies and mediate opsonophagocytosis against *A. pleuropneumoniae.*

## Conclusions

Strain SC105 (a representative of the dominant serotype of *H. parasuis* in the upper respiratory tract of swine) and strain SC-1 (a representative of popular pulmonary virulent serotype of *H. parasuis*) were selected in this study. We analyzed the differences in membrane proteomic level between SC105 and SC-1. Several proteins annotated by bioinformatic interpretation were proposed to be associated with bacterial adaptation to the upper respiratory tract and pathogenesis. Proteins overexpressed in strain SC105 enabled *H. parasuis* to colonize in the nutritionally deficient upper respiratory tract of swine. Following pathogenesis, several new proteins involved in enhancing bacterial defense and invasion capability, were found to be differentially expressed in strain SC-1 versus SC105. Our results provided further insight into the mechanism of colonization and pathogenesis of *H. parasuis*. With the increasing number of infections produced by *H. parasuis* and increasing antibiotic resistance among bacterial pathogens, we propose that pharmacological inhibition with the tonB-dependent siderophore receptor, KdsA and HtrA, as new therapeutic targets can be an effective alternative strategy for the clinical treatment of *H. parasuis*.

## Methods

### Ethics statement

Animal experiments were carried out in strict accordance with the recommendations of the China Regulations for the Administration of Affairs Concerning Experimental Animals 1988 and the Sichuan Regulations for the Administration of Affairs Concerning Experimental Animals 2012. All animal research protocols were approved by the Sichuan Agricultural University Institutional Animal Care and Use Committee. All animal experimental procedures have been refined to provide for maximum comfort and minimal stress to the animals.

### Bacterial strains and culture conditions

Two *H. parasuis* strains were used in this study. Strain SC-1 was isolated from the lung of a diseased piglet with fibrinous polyserositis in Sichuan province, China, in 2009. Strain SC105 was isolated from the nasal swab of a healthy pig also in Sichuan province, China, in 2010. The strains were identified using a specific PCR and biochemical test as previously described [72,73]. The serotypes of the two strains were then identified by a previously described method [7]. The strains were grown in Tryptic Soy Broth medium (Difco Laboratories, Detroit, USA) or maintained on Tryptic Soy agar (Difco Laboratories, Detroit, USA) at 37°C, both of which were added with 5% bovine serum and 0.01% NAD. Samples for proteome analysis and real-time PCR were collected at 0.5–0.6 optical density at 600 nm (OD_600_) of the cultures.

### Growth curve of the two strains

A single colony was transferred from Tryptic Soy agar into 5 mL of Tryptic Soy Broth medium for cultivation overnight at 37°C. The next day, 1 mL of initial culture was added to 100 mL of fresh Tryptic Soy Broth medium for subcultivation. To monitor the growth of bacterial cultures, the OD_600_ of the cultures was measured. Bacterial cultures were sampled at hourly intervals from the time of inoculation for a 20 h of inoculation.

### Challenge experiments in mice

A total of 110 female inbred BALB/c mice (West China School of Medicine, Sichuan University, China) aged 6 to 8 weeks were used. They were randomly allocated to 11 groups, with 10 mice per group. Five groups were intraperitoneally injected with SC-1, and the other five groups were challenged with SC105 at different doses (5 × 10^8^–2 × 10^9^ cfu per mouse). One group assigned to the control group was injected with PBS. Bacterial cultures were collected at OD_600_ = 0.5-0.6, washed twice in PBS, and adjusted to the appropriate doses. All mice were housed under comfortable conditions with food and water *ad libitum.* Clinical symptoms caused by intervention were monitored every 12 hours for 7 days post-infection. Once the mice^’^s spirits are drooping along with the back arched, rough fur and no eating or drinking, they were judged to death and euthanized by intraperitoneal injection of overdose (100 mg/kg) of pentobarbital per mouse out of humanitarian concerns.

### Protein extraction

To prepare membrane proteins, 300 mL cultures were harvested by centrifugation (7200 × *g* for 10 min at 4°C). The cells were washed three times with cold Tris–HCl (pH 9.5) plus protease inhibitor cocktail (Roche, Germany), resuspended in cold Tris–HCl (pH 9.5) containing protease inhibitor cocktail and sonicated on ice under the following conditions: 2 s of sonication with a 2 s interval set at 30% duty cycle. Every cycle lasts for 2 min with a 2 min interval. After the cells were completely split, the cell lysate was centrifuged at 15 000 × *g* for 20 min at room temperature to collect the insoluble pellet. The pellet was solubilized with lysis buffer (5 M urea, 2 M thiourea, 2% w/v CHAPS, 2% w/v SB 3–10, 40 mM Tris, and 2 mM TBP) after washing twice with cold Tris–HCl (pH 9.5). The mixture was vortexed for 5 min and centrifuged at 15 000 × *g* for 10 min at room temperature to pelletize the insoluble components. The supernatant was recovered, and the protein concentration was assayed with a Bio-Rad Protein Assay Kit I (Biorad, USA). Three separate biological replicates were performed for 2-DE analysis.

### 2-DE

In the 2-DE experiment, to ensure reproducibility, three technical replicates were performed for each biological replicate. IEF was performed using the PROTEAN IEF System (Biorad, USA) and Immobiline IPG strips (17 cm, pH 3–10; Biorad, USA). Then, 300 μg/strip prepared membrane proteins were mixed with rehydration solution (5 M urea, 2 M thiourea, 2% w/v CHAPS, 2% w/v SB 3–10, 40 mM Tris, 0.2% w/v Bio-Lyte 3/10 ampholyte, 2 mM TBP, and 0.002% w/v bromophenol blue) and loaded onto IPG strips. Strips were rehydrated at 50 V for 12 h and then sequentially focused at 200 V for 30 min, 500 V for 30 min, 1000 V for 1 h, 10 000 V for 5 h, and finally 10 000 V for 60 kVh. After IEF, the IPG strips were equilibrated in equilibration buffer I (2% w/v DTT, 2% w/v SDS, 6 M urea, 20% (v/v) glycerol, and 0.375 M Tris–HCl; pH 8.8) and equilibration buffer II (2.5% w/v iodoacetamide, 2% w/v SDS, 6 M urea, 20% (v/v) glycerol, and 0.375 M Tris–HCl; pH 8.8) for 15 min. After equilibration, The IPG strips were applied onto 10% SDS-polyacrylamide gels using PROTEAN® II xi (Biorad, USA). Separation on the second dimension was performed at 80 V for 30 min followed by 200 V for 4 h. The gels were visualized with staining solution (34% methanol, 17% ammonium sulfate, 0.1% CBB G-250, 3% phosphoric acid) and washed with deionized water. Gel scanning and data analysis were carried out using PDQuest 8.0 software (Biorad, USA).

### Protein identification by MS

Protein spots of interest were excised from the gels and detained three times with 50 μL of 25 mM ammonium bicarbonate/50% acetonitrile for 20 min at 37°C, shrunk with 50 μL of 100% acetonitrile, and then vacuum dried for 10 min. Every protein spot was reswollen with 3 μL of 0.01 mg/ml trypsin in 25 mM ammonium bicarbonate at 4°C for 30 min followed by in-gel tryptic degradation at 37°C for 16 h. The supernatant was mixed with an equal volume of MALDI matrix (10 mg/ml α-cyano-4-hydroxycinnamic acid saturated solution with 50% acetonitrile in 0.1% trifluoroacetic acid).

The sample solution was transferred to a MALDI target disk, dried at room temperature, and analyzed by MALDI-TOF/TOF using a 4800 Proteomics Analyzer (Applied Biosystems). This procedure was carried out in positive ion reflector mode with a mass range of 800–4000 Da at an accelerating voltage of 20 kV. Each spectrum was calibrated with a peptide standard kit (Applied Biosystems). For each sample spot, the seven most intense parent mass peaks with a minimum signal-to-noise ratio of 50 were selected for further tandem TOF/TOF analysis.

### Bioinformatics analysis

MS and MS/MS data were processed using Global Proteome Server Explorer 3.6 (Applied Biosystems). MASCOT was used to search the data against the NCBInr protein database. Species selection was limited to *H. parasuis*. For all identified proteins, the MASCOT score (*p* < 0.05) was considered significant. These data were submitted to SignalP 3.0, LipoP 1.0, and SecretomeP 2.0 software for prediction of signal peptides, potential lipoproteins, and signal peptide-independent protein secretion, respectively. The software TMHMM 2.0 and PSORT Version 3.0 were separately used to predict transmembrane domains and cellular localizations. Protein functions were assigned using Tigrfam and BLASTp [74].

### Quantitative real-time PCR (qRT-PCR) for gene expression verification

Four selected proteins were further analyzed for changes in mRNA levels by qRT-PCR. The 16 s rRNA gene selected as a reference housekeeping gene was used as an internal standard in PCR amplification. Total RNA was extracted from different *H. parasuis* strains using an RNAprep Pure Cell/Bacteria Kit (Tiangen Biotech, China). Reverse transcription of total RNA was performed with a PrimeScript^TM^ RT Reagent Kit (TaKaRa Biotech). Quantitative real-time PCR was then performed using the MiniOpticon™ Real-Time PCR Detection System (Bio-Rad, USA). Primers were designed with the software Primer Premier 5.0 (Table 3). Melting curves were carefully monitored to avoid nonspecific amplifications.

**Table 3:** Primer sequences for real time PCR

**Gene**	**Primer sequence**	**Size (bp)**
16 s rRNA sense primer	5′ CGGGAAACTGTCGCTAAT 3′	160
16 s rRNA anti-sense primer	5′ TGTGGCTGGTCATCCTCT 3′	
espP sense primer	5′ GCAGCACTCACTTCTCAGC 3′	205
espP anti-sense primer	5′ CACCGTCAACGGCATAG 3′	
hbpA sense primer	5′ CCAAATCACCCATACCAC 3′	160
hbpA anti-sense primer	5′ CCATACCTAAACTCGCTAAG 3′	
htrA sense primer	5′ TGTAAACGGACAAGCATT 3′	178
htrA anti-sense primer	5′ AAACTGACGAGGAGAACC 3′	
potD sense primer	5′ TGACCCAAGCACGATTAC 3′	271
potD anti-sense primer	5′ TGCCGAACCTGTCCATT 3′	

## Competing interest

The authors declare that they have no competing interests.

## Authors’ contributions

LhZ and YpW are principal investigators, designed and conceived the experiments, and prepared the manuscript. YL and XlW carried out two-dimensional gel electrophoresis. XfY provided membrane proteomes. XtW, RW and XbH participated in the design of the study and performed the bioinformatics analysis. YH, QgY and MfL helped to draft the manuscript. SjC designed and conceived the experiments and prepared the manuscript. All authors read and approved the final manuscript.
